# Joint *k*-*ω* Space Image Reconstruction and Data Fitting for Chemical Exchange Saturation Transfer Magnetic Resonance Imaging

**DOI:** 10.3390/tomography10070085

**Published:** 2024-07-15

**Authors:** Yuting Peng, Yan Dai, Shu Zhang, Jie Deng, Xun Jia

**Affiliations:** 1 Department of Radiation Oncology and Molecular Radiation Sciences, Johns Hopkins University, Baltimore, MD 21287, USA; 2 Department of Radiation Oncology, University of Texas Southwestern Medical Center, Dallas, TX 75390, USA; 3 Department of Radiology, Houston Methodist Research Institute, Houston, TX 77030, USA

**Keywords:** CEST, jointreconstruction, data fitting, Z-spectra

## Abstract

Chemical exchange saturation transfer (CEST) magnetic resonance imaging (MRI) is a novel MRI technology to image certain compounds at extremely low concentrations. Long acquisition time to measure signals at a set of offset frequencies of the Z-spectra and to repeat measurements to reduce noise pose significant challenges to its applications. This study explores correlations of CEST MR images along the spatial and Z-spectral dimensions to improve MR image quality and robustness of magnetization transfer ratio (MTR) asymmetry estimation via a joint *k*-ω reconstruction model. The model was formulated as an optimization problem with respect to MR images at all frequencies ω, while incorporating regularizations along the spatial and spectral dimensions. The solution was subject to a self-consistency condition that the Z-spectrum of each pixel follows a multi-peak data fitting model corresponding to different CEST pools. The optimization problem was solved using the alternating direction method of multipliers. The proposed joint reconstruction method was evaluated on a simulated CEST MRI phantom and semi-experimentally on choline and iopamidol phantoms with added Gaussian noise of various levels. Results demonstrated that the joint reconstruction method was more tolerable to noise and reduction in number of offset frequencies by improving signal-to-noise ratio (SNR) of the reconstructed images and reducing uncertainty in MTR asymmetry estimation. In the choline and iopamidol phantom cases with 10.5% noise in the measurement data, our method achieved an averaged SNR of 31.0 dB and 32.2 dB compared to the SNR of 24.7 dB and 24.4 dB in the conventional reconstruction approach. It reduced uncertainty of the MTR asymmetry estimation over all regions of interest by 54.4% and 43.7%, from 1.71 and 2.38 to 0.78 and 1.71, respectively.

## 1. Introduction

Chemical exchange saturation transfer (CEST) magnetic resonance imaging (MRI) is an important MRI technique that allows for non-invasive visualization of the exchange of protons between water and other molecules in biological tissues. The CEST contrast mechanism is based on the transfer of magnetization from exchangeable protons to water, which can be detected using standard magnetic resonance pulse sequences. CEST MRI generally involves the acquisition of a saturation spectrum or Z-spectrum, in which a series of data are acquired using radio-frequency (RF) saturation with varying offset frequencies. After reconstructing the images at each frequency ω, the ratio of saturated ($S_{sat}$) and unsaturated ($S_{0}$) signals is plotted as a function of ω. Because the exchangeable protons tend to resonate downfield (at higher frequency) from water and in an effort to remove the symmetric effects from direct water saturation, CEST data are commonly analyzed using asymmetry analysis with respect to the water frequency set at $\omega_{1}=0$ and normalized to the unsaturated signal ($S_{0}$). The asymmetry analysis of the Magnetization Transfer Ratio (MTR) is defined as:

(1) $$\begin{array}{c} MTR_{asym}\left(\omega \right)=MTR\left(\omega \right)-MTR\left(-\omega \right)=\left[S_{sat}\left(-\omega \right)-S_{sat}\left(\omega \right)\right]/S_{0}. \end{array}$$

This approach provides a sensitive and specific means of detecting tissue properties and pathological conditions that may not be observable using conventional MRI [1,2,3]. One of the most promising applications of CEST MRI is its ability to detect low-concentration metabolites, such as creatine, glutamate, and lactate. This is of vital importance for medicine, as these metabolites play critical roles in various biological processes and are implicated in numerous diseases. Having been applied in a wide range of clinical scenarios [4], CEST MRI has been shown to provide useful information for the diagnosis of numerous human diseases, including tumors [5,6], strokes [7,8], neurological disorders [9,10], lymphedema [11], osteoarthritis [12,13], ischemia [14,15], and epilepsy [16].

Despite its promising potential, CEST MRI encounters several technical challenges that limit its clinical use. One of the major challenges is the long acquisition time due to measurements at a set of offset frequencies ω of the Z-spectrum [17]. Sometimes signal-to-noise ratio (SNR) is sacrificed to reduce acquisition time, which affects accuracy of images and reliability of derived information [4,18,19]. There is a desire to develop new reconstruction algorithms for CEST MRI to obtain MRI images with high quality and accuracy.

Generally, MRI reconstruction can be taken as an inverse problem that aims to rebuild data from non-ideal measurements. The conventional CEST MRI reconstruction method includes a two-step process in which the magnitude CEST MRI image acquired at each offset frequency is reconstructed from its own k-space data, for example, by Fast Fourier Transform (FFT). This is followed by a pixel-by-pixel fitting of the CEST MRI signal to obtain the Z-spectrum. Model-based nonlinear approaches have been developed to exploit the fact that a steady state solution of the Bloch McConnell equation yields a Lorentzian distribution in the spectral dimension [20,21,22,23]. The measured Z-spectra were delineated by combining multiple shifted Lorentzian functions that correspond to different pools of interest. A model-based CEST MRI for robust Z-spectrum analysis was proposed in which the spectra of interest were directly estimated from the measurements by incorporating subspace-based spectral signal decomposition into the CEST MRI measurement model [24]. To explore image domain correlation, compressed sensing has been used to regulate image properties, allowing for CEST reconstruction with better image quality [25,26]. Recently, with the advancements in deep learning in medical imaging [27,28], data-driven approaches have been introduced to MRI reconstruction [29]. In the CEST MRI context, deep neural networks (DNNs) were used to predict model fitting parameters [30] or to directly reconstruct images [31]. Using a variational network, a model-based DNN was developed for fast multi-coil CEST imaging, which took advantage of the redundancy in neighboring CEST frames and correlation in the spatial-frequency domain [32]. A sequence-to-sequence (seq2seq) framework was recently employed to recover dense CEST Z-spectra from experimentally acquired images at sparse frequency [33].

For each reconstruction approach, a certain type of prior information is incorporated to compensate for the missing or low-quality information in the measurement. In this work, based on the prior knowledge of the number of exchanging pools and the corresponding frequency shift of each pool, we proposed a novel joint *k*-ω CEST MRI reconstruction algorithm. The method falls into the conventional model-based approaches, with the explicit use of the relationship between the CEST MRI signals at offset frequencies on a pixel-by-pixel basis as a constraint. During the reconstruction iterations, the error in one MR image at a certain frequency can be mitigated by images at other offset frequencies. In addition, image-domain regularization in the CEST MR images was considered a priori by using an image denoising filter algorithm via the plug-and-play (PnP) approach [34]. Compared to the data-driven deep learning approaches, our model-driven method avoids model training and issues such as data quality and domain variation, and holds the advantages of interpretability.

## 2. Materials and Methods

### 2.1. Model for Joint Reconstruction and Data Fitting

Let us denote the measured complex k-space data at offset frequencies ω as g(ω). The complex MR images to be reconstructed include the (real) amplitude component f(x,ω) and the (complex) phase factor image A(x,ω). $A\left(x,\omega \right)=e^{i\phi (x,\omega )}$, where ϕ(x,ω) is the phase image for each frequency ω. These terms are related by a Fourier transform model

(2) g(ω)=FA(x,ω)f(x,ω)+n,

where *n* denotes noise signal in data acquisition. F is the Fourier transform operator. The MR images at different offset frequencies ω are related by the Z-spectrum as follows:

(3) $$f\left(x,\omega \right)=f\left(x,\omega_{0}\right)Z\left(x,\omega \right),$$

where $f(x,\omega_{0})$ is the reference signal intensity acquired without radio-frequency wave (RF) saturation. Z(x,ω) is the so-called Z-spectrum, describing the attenuation of the water signal versus the offset frequencies.

We proposed to solve the optimization model to jointly estimate the image f(x,ω), A(x,ω), and the Z-spectrum Z(x,ω) as follows:

(4) $$\begin{array}{cc} \left\{f^{*},A^{*},Z^{*}\right\} & =\underset{\left\{f,A,Z\right\}}{\mathrm{argmin}}\frac{1}{2}\sum_{\omega }{\left|FA(x,\omega )f(x,\omega )-g(\omega )\right|}^{2}+R\left[f\left(x,\omega \right),\lambda \right],\\ \; & s.t.\;f\left(x,\omega \right)=f\left(x,\omega_{0}\right)Z\left(x,\omega \right). \end{array}$$

In this optimization problem, the first term in the objective function was a data fidelity term that ensures the agreement between the reconstructed images and the measurements g(ω). The term *R* denotes the image domain regularization functions on MR images, and the constraint enforces the relationship posed by the Z-spectra for each pixel. λ was the parameter in the regularization function to govern its strength.

Generally speaking, the regularization term can be chosen based on specific assumptions of the solution’s properties in the spatial domain. In this study, the term *R* was taken as the block matching and 3D filtering method (BM3D) [35], which will be employed via the plug-and-play approach presented in the next subsection. But we remark that our approach is generally applicable to other regularization terms, which will be discussed later. As for the constraint shown in Equation (3), this was achieved by enforcing the function Z(x,ω) in a specific form with multiple components corresponding to different pools to be probed in CEST MRI. While the shape of the peaks are generally expected to be Lorentzian functions, we empirically tested Lorentzian and Gaussian fitting and found that a combined fitting approach with the Lorentzian function for the resonance frequency $\omega_{1}$ of the water pool and Gaussian function for other frequencies of other pools worked the best for our experimental data, namely

(5) $$Z\left(x,\omega \right)=1-\left[\frac{a(x)}{{\left(\omega -\omega_{1}\right)}^{2}+\Gamma \left(x\right)}+\sum_{i=2}b_{i}\left(x\right)\exp[-\frac{{\left(\omega -\omega_{i}\right)}^{2}}{\sigma_{i}{\left(x\right)}^{2}}]\right],$$

where $\omega_{1}$ and $\omega_{i}$ for i=2,… were assumed to be known based on specific experimental setup. Detailed justification of this choice will be presented in Section 3.1.

### 2.2. Numerical Algorithm and Implementation

The optimization problem in Equation (4) is complicated with respect to the independent variables. We solved the problem via an iterative algorithm. Since the amplitude f(x,ω) and phase image A(x,ω) are multiplied together, we empirically estimated the solution for the phase image A(x,ω) during the iterative process. The specific method will be presented below. Once this term was estimated, we fixed it and used the alternating direction method of multipliers (ADMM) [36] to solve the optimization problem with respect to *f* and *Z* while incorporating the regularization approach via the PnP framework [34].

As such, we first considered the optimization problem equivalent to Equation (4) by introducing two auxiliary variables *v* and *T*:

(6) $$\begin{array}{cc} \left\{f,Z,v,T\right\} & =\underset{\left\{f,Z,v,T\right\}}{\mathrm{argmin}}\frac{1}{2}\sum_{\omega }{\left|FA(x,\omega )f(x,\omega )-g(\omega )\right|}^{2}+R\left[v\left(x,\omega \right),\lambda \right],\\ \; & s.t.\;f\left(x,\omega \right)=f\left(x,\omega_{0}\right)T\left(x,\omega \right),\;v\left(x,\omega \right)=f\left(x,\omega \right),\;T\left(x,\omega \right)=Z\left(x,\omega \right). \end{array}$$

The augmented Lagrangian of this problem is

(7) $$\begin{array}{cc} L_{\rho } & =\frac{1}{2}\sum_{\omega }{\left|FA(x,\omega )f(x,\omega )-g(\omega )\right|}^{2}+R\left[v\left(x,\omega \right),\lambda \right]\\ \; & +\sum_{\omega }y_{\omega }^{T}\left[f\left(x,\omega \right)-f\left(x,\omega_{0}\right)T\left(x,\omega \right)\right]+\sum_{\omega }\frac{\rho }{2}{\left|f\left(x,\omega \right)-f\left(x,\omega_{0}\right)T\left(x,\omega \right)\right|}^{2}\\ \; & +\sum_{\omega }z_{\omega }^{T}\left[v\left(x,\omega \right)-f\left(x,\omega \right)\right]+\sum_{\omega }\frac{\rho }{2}{\left|v\left(x,\omega \right)-f\left(x,\omega \right)\right|}^{2}\\ \; & +t_{\omega }^{T}\left[T\left(x,\omega \right)-Z\left(x,\omega \right)\right]+\frac{\rho }{2}{\left|T\left(x,\omega \right)-Z\left(x,\omega \right)\right|}^{2}, \end{array}$$

where $y_{\omega }$, $z_{\omega }$, $t_{\omega }$, and ρ are variables introduced in the algorithm. The key idea of the use of ADMM to solve the optimization problem is to iteratively address each variable. Specifically, it performs the following steps with the superscript *k* being the index of iteration:

(8) $$\begin{array}{cc} f^{k+1}\left(x,\omega \right) & =\underset{f(x,\omega )}{\mathrm{argmin}}\frac{1}{2}{\left|FA(x,\omega )f(x,\omega )-g(\omega )\right|}^{2}+y_{\omega }^{T}\left[f\left(x,\omega \right)-f^{k}\left(x,\omega_{0}\right)T^{k}\left(x,\omega \right)\right]\\ \; & +\frac{\rho }{2}{\left|f\left(x,\omega \right)-f^{k}\left(x,\omega_{0}\right)T^{k}\left(x,\omega \right)\right|}^{2}+z_{\omega }^{T}\left[v^{k}\left(x,\omega \right)-f\left(x,\omega \right)\right]\\ \; & +\frac{\rho }{2}{\left|v^{k}\left(x,\omega \right)-f\left(x,\omega \right)\right|}^{2}, \end{array}$$

for $\omega \neq \omega_{0}$. For $\omega_{0}$:

(9) $$\begin{array}{cc} f^{k+1}\left(x,\omega_{0}\right) & =\underset{f(x,\omega_{0})}{\mathrm{argmin}}\frac{1}{2}{\left|FA\left(x,\omega_{0}\right)f\left(x,\omega_{0}\right)-g\left(\omega_{0}\right)\right|}^{2}\\ \; & +\sum_{\omega \neq \omega_{0}}y_{\omega }^{T}\left[f^{k}\left(x,\omega \right)-f\left(x,\omega_{0}\right)T^{k}\left(x,\omega \right)\right]\\ \; & +\sum_{\omega \neq \omega_{0}}\frac{\rho }{2}{\left|f^{k}\left(x,\omega \right)-f\left(x,\omega_{0}\right)T^{k}\left(x,\omega \right)\right|}^{2}+z_{\omega }^{T}\left[v^{k}\left(x,\omega_{0}\right)-f\left(x,\omega_{0}\right)\right]\\ \; & +\frac{\rho }{2}{\left|v^{k}\left(x,\omega_{0}\right)-f\left(x,\omega_{0}\right)\right|}^{2}. \end{array}$$

As for the variables *Z*, *v*, and *T*, for each individual ω:

(10) $$\begin{array}{c} Z^{k+1}\left(x,\omega \right)=\underset{Z(x,\omega )}{\mathrm{argmin}}t_{\omega }^{T}\left[T^{k}\left(x,\omega \right)-Z\left(x,\omega \right)\right]+\frac{\rho }{2}{\left|T^{k}\left(x,\omega \right)-Z\left(x,\omega \right)\right|}^{2}, \end{array}$$

(11) $$\begin{array}{cc} v^{k+1}\left(x,\omega \right) & =\underset{v(x,\omega )}{\mathrm{argmin}}R\left[v\left(x,\omega \right),\lambda \right]+z_{\omega }^{T}\left[v\left(x,\omega \right)-f^{k}\left(x,\omega \right)\right]\\ \; & +\frac{\rho }{2}{\left|v\left(x,\omega \right)-f^{k}\left(x,\omega \right)\right|}^{2}, \end{array}$$

(12) $$\begin{array}{cc} T^{k+1}\left(x,\omega \right) & =\underset{T(x,\omega )}{\mathrm{argmin}}y_{\omega }^{T}\left[f\left(x,\omega \right)-f\left(x,\omega_{0}\right)T\left(x,\omega \right)\right]\\ \; & +\frac{\rho }{2}{\left|f\left(x,\omega \right)-f\left(x,\omega_{0}\right)T\left(x,\omega \right)\right|}^{2}+t_{\omega }^{T}\left[T\left(x,\omega \right)-Z^{k}\left(x,\omega \right)\right]\\ \; & +\frac{\rho }{2}{\left|T\left(x,\omega \right)-Z^{k}\left(x,\omega \right)\right|}^{2}. \end{array}$$

Finally, the variables were updated as

(13) $$\begin{array}{c} y_{\omega }^{k+1}=y_{\omega }^{k}+\rho \left[f^{k}\left(x,\omega \right)-f^{k}\left(x,\omega_{0}\right)Z^{k}\left(x,\omega \right)\right], \end{array}$$

(14) $$z_{\omega }^{k+1}=z_{\omega }^{k}+\rho \left[v^{k}\left(x,\omega \right)-f^{k}\left(x,\omega \right)\right],$$

(15) $$\begin{array}{c} t_{\omega }^{k+1}=t_{\omega }^{k}+\rho \left[T^{k}\left(x,\omega \right)-Z^{k}\left(x,\omega \right)\right]. \end{array}$$

The first two subproblems in Equations (8) and (9) with respect to images f(x,ω) at various offsets ω were quadratic optimization problems and were solved using the conjugate gradient method for least squares (CGLS) algorithm. While the term f(x,ω) is supposed to be real, generally speaking, a complex solution would be obtained after solving the equations. We hence singled out the phase factor and combined that with the A(x,ω) term to update the estimated phase factor images.

The sub-problem in Equation (10) with respect to Z(x,ω) was essentially data fitting to determine the Z-spectra by fitting data pixel-wise in Equation (5) form. This was achieved by enforcing the function Z(x,ω) as a sum of multiple peaks corresponding to different pools to be probed. Empirically, this fitting approach can further reduce the noise perturbation during the iterative optimization process.

The sub-problem in Equation (11) was an image domain processing problem, which estimates a solution with an enhanced quality based on the regularization term for image $f\left(x,\omega \right)-\frac{z_{\omega }^{T}}{\rho }$. We employed the PnP approach, which plugs in a powerful image denoising algorithm in its place. Previous studies have empirically demonstrated the validity of this approach in terms of producing high-quality results in various applications. In this study, we used the BM3D denoising method in this PnP framework, but the algorithm is applicable to other denoising methods, including deep learning-based ones. This step is symbolically denoted as

(16) $$v^{k+1}\left(x,\omega \right)=BM3D\left[f^{k}\left(x,\omega \right)-\frac{z_{\omega }^{T}}{\rho },\lambda \right].$$

The BM3D denoising operation requires a hyper-parameter λ, which specifies the noise variance. To estimate the λ for the images $f^{k}\left(x,\omega \right)-\frac{z_{\omega }^{T}}{\rho }$, we first estimated local noise variance of patches with a size of 5 × 5 pixels, and set λ as half of the mode value of local noise variance values. This choice led to a conservative estimation to avoid over-smoothing. As described above, we employed denoising approaches both in the spatial image and Z-spectrum domains, further improving the CEST MRI reconstruction performance.

Finally, for Equation (12), it has a closed form solution as follows:

(17) $$T^{k+1}\left(x,\omega \right)=\frac{\rho Z\left(x,\omega \right)-t_{\omega }^{T}+f\left(x,\omega_{0}\right)y_{\omega }^{T}+\rho f\left(x,\omega \right)f\left(x,\omega_{0}\right)}{\rho f{\left(x,\omega_{0}\right)}^{2}+\rho }.$$

**Figure 1:**
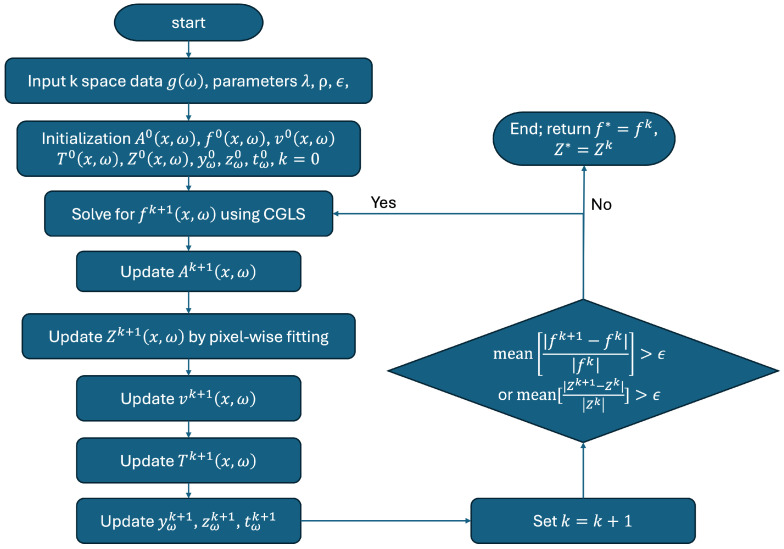
Flowchart illustrating the iterative process of CEST MRI joint reconstruction.

The iterative process in Equations (8)–(15) continued until convergence, as indicated by the stopping criteria that the mean relative differences of f(x,ω) and the Z-spectra Z(x,ω) in two successive iteration steps were less than a threshold ϵ=0.001. The algorithm used to solve the joint reconstruction and data fitting problem is summarized in the workflow shown in Figure 1.

The algorithm was implemented in Matlab 2023b, and computations were performed on a workstation with an Intel(R) Xeon(R) Silver 4214 CPU of 2.40 GHz frequency. We empirically set the parameter ρ=0.5.

### 2.3. Evaluations

**Figure 2:**
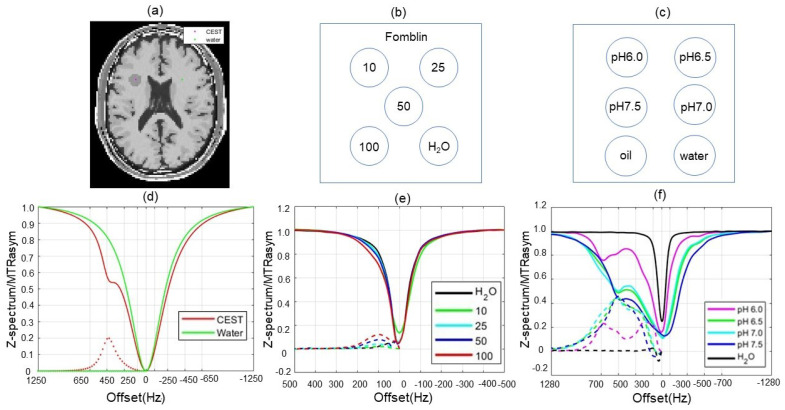
Illustration for the (**a**) two-compartment numerical phantom, (**b**) the experimental two-compartment phantom which contained choline water solutions with concentrations: 10, 25, 50, and 100 mM in the Fomblin background, and (**c**) the experimental multi-compartment phantom which contained iopamidol (IsOvue-300, Bracco Diagnostics, Milan, Italy) solutions at pH 6.0, 6.5, 7.0, and 7.5. (**d**–**f**) Z-spectra (solid lines) and corresponding MTR asymmetry (dotted lines) for the phantom in (**a**–**c**), respectively.

A brain phantom simulated by CEST MRI with two compartments (Figure 2a) and the corresponding spectra was generated using a pool of water and a CEST region of interest (ROI). We first created a phantom using the MRiLab tool [37], then inserted the ROI. For the CEST pool, the proton density (PD) was set as 0.85, T1/T2=1200/120 ms, proton exchange rate with the water pool resonance frequency $\omega_{2}=450$ Hz. For the water pool, $\omega_{1}=0$ Hz; proton density and T1/T2 relaxation times varied with tissues in brain phantom as reported in the MRiLab simulation. The MR image with a matrix of 128 × 128 for offset frequencies from −1250 to 1250 Hz with a frequency step of 50 Hz was created as the GT images. $\omega_{0}=-1250$ Hz. A smoothly varying phase image was created. K-space data g(ω) of the corresponding CEST MR images were generated by FFT.

Our reconstruction algorithm was further evaluated semi-realistically using an approach with experimental phantoms and simulated k-space data. CEST MRI data sets were acquired on a Philips 3T Ingenia MRI scanner (Philips Healthcare, Andover, MA, USA) for two phantoms. An experimental phantom shown in Figure 2c contained choline water solutions with various concentrations (10, 25, 50, and 100 mM) in the Fomblin background. The Z-spectra and the corresponding MTR asymmetry plots were acquired experimentally, shown in Figure 2d for the two pools. Based on the image and Z-spectra acquired experimentally, we generated data g(ω) used for reconstruction. In this case, $\omega_{0}$ = 500 Hz, $\omega_{1}$ = 0 Hz, and $\omega_{2}$ = 130 Hz. The other experimental phantom shown in Figure 2e included a water background and iopamidol (IsOvue-300, Bracco Diagnostics, Milan, Italy) solutions at pH 6.0, 6.5, 7.0, and 7.5. The Z-spectra and the corresponding MTR asymmetry plots are presented in Figure 2f for different iopamidol solutions. We generated g(ω) in the same way as described above. $\omega_{0}=-1250$ Hz. In this case, in addition to the resonance frequency $\omega_{1}=0$ Hz, we considered two other frequencies: $\omega_{2}=530$ Hz and $\omega_{3}=700$ Hz.

To evaluate the performance of the proposed joint *k*-ω reconstruction method and compare it with that of the conventional FFT-based reconstruction method, Gaussian white noises with various amplitudes (0.5∼40% relative to the mean signal of the k-space data for MR images at offset frequency $\omega_{0}$) were added to the real and imaginary parts of the k-space data. Signal-to-noise ratio (SNR) of the reconstructed images were computed, defined as the ratio between the mean signal of ROI and the standard deviation of the background noise. We further computed the averaged SNR over all MR images of different offset frequencies as a metric of image quality and compared the values of this metric to the results of our algorithm and the conventional algorithm using FFT-based image reconstruction, followed by pixel-wise Z-spectrum fitting. Additionally, the MTR asymmetry map was calculated for the reconstruction results by determining the MTR asymmetry values at the offset frequency $\omega_{i}$ for each pixel located.

One potential advantage of including the correlation in the spectral space is to serve as a regularization in the frequency direction to enable a reduction in the number of measurements. To evaluate the advantage of our algorithm in this regard, we performed studies with a reduced number of offset frequencies. Specifically, in the simulation case, we investigated algorithm performance using frequency values of 37 and 44 (15.6% and 27.5% reductions, respectively), as compared to the previous study with 51 frequency values.

## 3. Results

### 3.1. Choice of Fitting Functions

**Figure 3:**
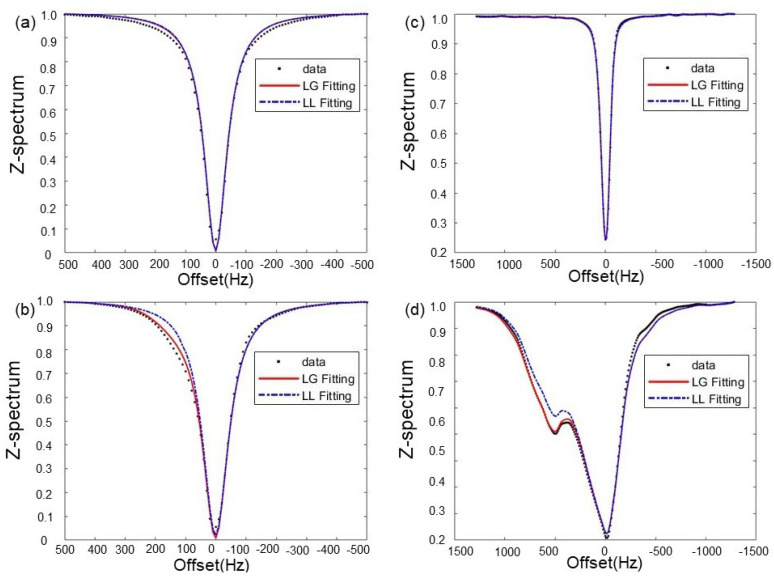
LGfitting (solid red line) and LL fitting (blue dashed line) for (**a**,**b**) the choline phantom and (**c**,**d**) the iopamidol phantom, respectively. The figures in the upper row (**a**,**c**) are water pools and those in the lower row (**b**,**d**) are CEST pools, respectively.

In Figure 3, we demonstrated the performance of the combined Lorentzian and Gaussian (LG) fitting on the two experimental datasets studied in this work, as compared to the conventional Lorentzian (LL) fitting. It was empirically found that the LG fitting achieved better results than the LL fitting. The mean absolute error (MAE) relative to the GT data was calculated to represent the fitting performance. MAE of the LG fitting for the cases in Figure 3a–d were $1.30\times 10^{-2}$, $9.70\times 10^{-3}$, $1.40\times 10^{-3}$, and $1.12\times 10^{-2}$, while those of LL fitting were $1.36\times 10^{-2}$, $1.78\times 10^{-2}$, $1.60\times 10^{-3}$, and $2.10\times 10^{-2}$, respectively. The LG fitting reduced MAE on average by 27.3% in these cases compared to the LL fitting.

### 3.2. Numerical Simulations

**Figure 4:**
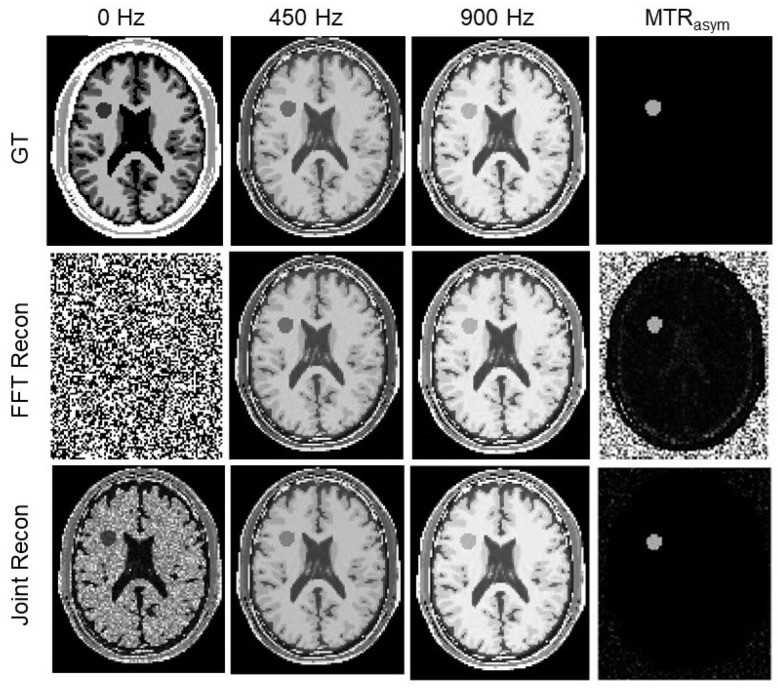
GT MR images, MR images reconstructed by the FFT-based algorithm and our algorithm, and MTR asymmetry map at $\omega_{2}$ = 450 Hz for the simulated phantom case with a noise level of 4.5%. The image windows at 0 Hz are [0 $5\times 10^{-6}$], [0 0.05] at 450∼900 Hz, and [0 0.2] for MTR asymmetry map.

**Figure 5:**
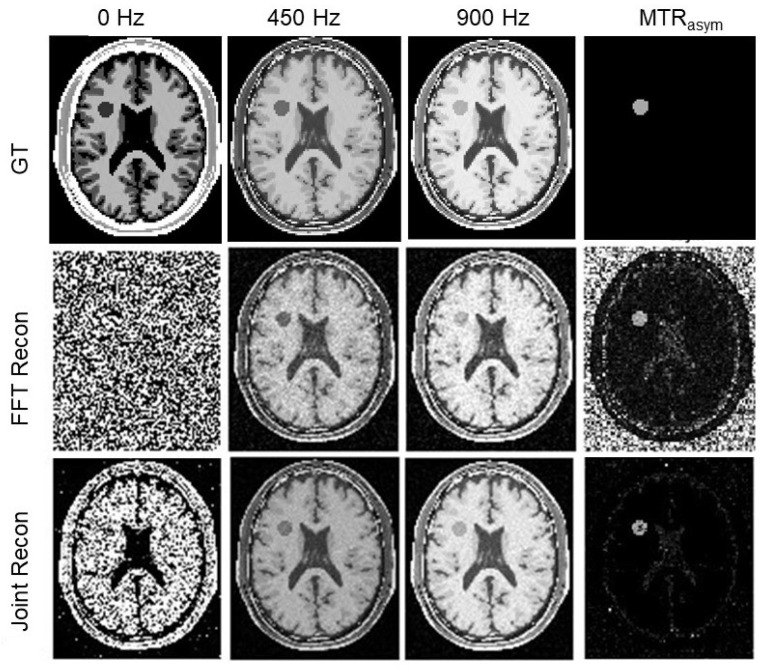
GT MR images, MR images reconstructed by the FFT-based algorithm and our algorithm, and MTR asymmetry map at $\omega_{2}$ = 450 Hz for the simulated phantom case with a noise level of 25%. The image windows at 0 Hz are [0 $5\times 10^{-6}$], [0 0.05] at 450∼900 Hz, and [0 0.2] for MTR asymmetry map.

**Figure 6:**
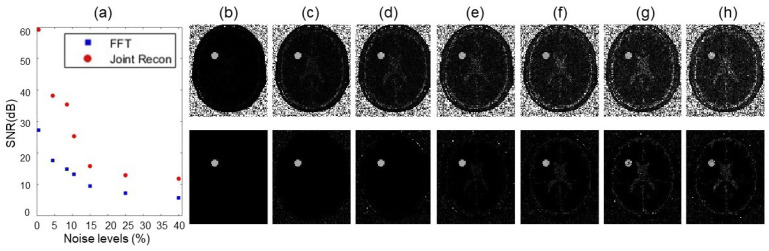
(**a**) SNR of reconstructed MR images as a function of noise levels. (**b**–**h**) MTR asymmetry maps at $\omega_{2}$ = 450 Hz by the conventional algorithm (upper row) and our algorithm (lower row) for the simulated phantom with various noise levels of 0.5%, 4.5%, 8.5%, 10.5%, 15%, 25%, and 40%, respectively. The display window of MTR asymmetry map is [0 0.2].

In Figure 4 and Figure 5, we presented GT images of the simulated phantom, reconstructed MR images by the FFT-based algorithm, and our algorithm for the two cases with added noise levels of 4.5% and 25%, respectively. In each case, we showed the MR images at offset frequencies of z = 0, 450, and 900 Hz, and the MTR asymmetry maps, respectively. We observed that SNRs of the results from our method improved significantly. Specifically, as shown in Figure 6a, SNRs for the MR images from the conventional method were in the range 5.7∼27.2 dB, while those for our algorithm were 11.7∼59.0 dB, approximately double the values of the conventional algorithm.

In Figure 6b–h, we presented the MTR asymmetry maps for the case with various noises levels of 0.5∼40%. The results demonstrated improvement in the prediction of MTR asymmetry analysis. While both methods gave similar results at the small noise limit, on the large noise end with 25∼40% noise levels, our algorithm delivered more robust MTR asymmetry maps than the conventional method.

### 3.3. Results of Choline Phantom Case

**Figure 7:**
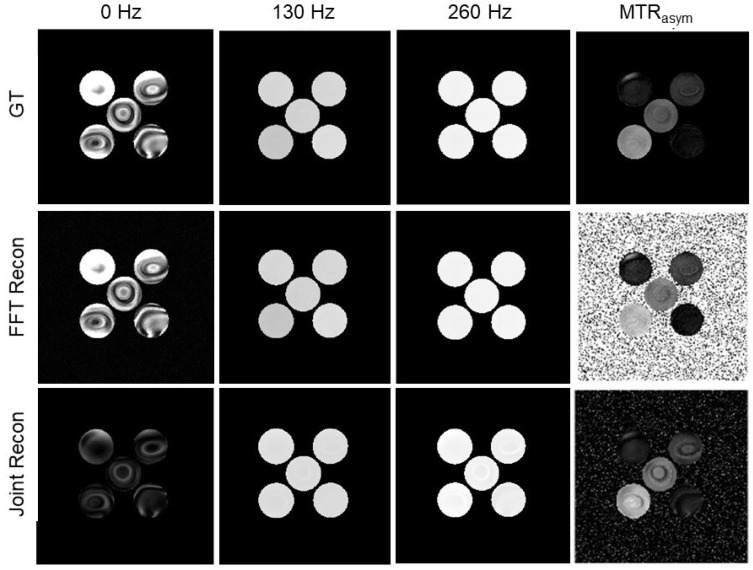
GT MR images, MR images reconstructed by the FFT-based algorithm and our algorithm, and MTR asymmetry map at $\omega_{2}$ = 130 Hz of the choline phantom with noise level 15% at offset frequencies ω = 0, 130, and 260 Hz. Image windows at 0 Hz are [0 0.1] and [0 1] at 130∼260 Hz. MTR asymmetry window is [0 0.2].

In Figure 7, we presented the GT images, the MR images reconstructed by the conventional method and our methods of the choline phantom with a noise level of 15%, as well as the MTR asymmetry map. Note that the signal and SNR of the reconstructed image at 0 Hz by our algorithm was lower than that of the conventional method due to the strong constraint on the Z-spectrum value at this frequency. However, the averaged SNR of the MR images over all frequencies were improved from 24.7 dB using the conventional method to 31.3 dB using our algorithm. Overall, as the noise levels were in the range 0.5∼40%, the averaged SNR of the CEST MR images were in the range 16.3∼46.4 dB, and our algorithm increased the averaged SNR to 21.6∼60.0 dB.

**Figure 8:**
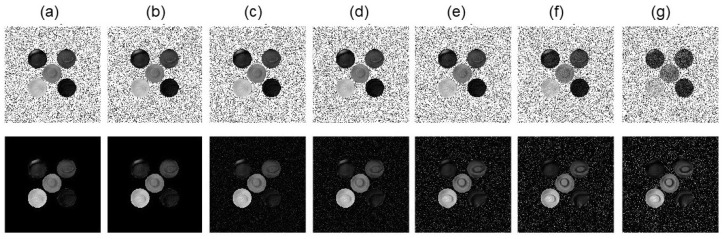
MTR asymmetry maps at $\omega_{2}$ = 130 Hz of conventional algorithm (upper row) and our algorithm (lower row) of the experimental choline phantom with various noise levels (**a**) 0.5%, (**b**) 2.5%, (**c**) 4.5%, (**d**) 10.5%, (**e**) 15%, (**f**) 25%, and (**g**) 40%, respectively. MTR asymmetry map window is [0 0.2].

**Figure 9:**
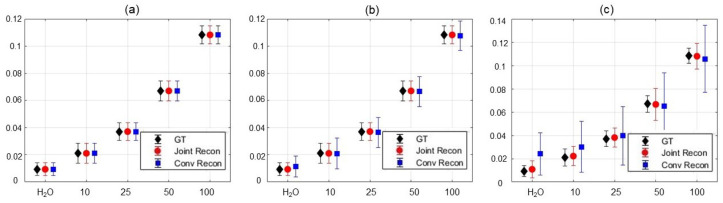
Mean and standard deviation of MTR asymmetry in each ROI at $\omega_{2}$ = 130 Hz for water and choline concentrations of 10, 25, 50, and 100 mM of the GT; results reconstructed by the conventional method and our method with noise levels of (**a**) 0.5%, (**b**) 4.5%, and (**c**) 10.5%, respectively.

In Figure 8, we presented the MTR asymmetry maps for the case with various noise levels in the range 0.5∼40%. We further present the MTR asymmetry analysis at 130 Hz for water and choline at various concentrations in Figure 9. The error bar shows the standard deviation of MTR asymmetry within each ROI. In the case of low noise, both the conventional method and our method performed similarly. However, as the noise level increased, our method was found to be more tolerable to noise than the conventional method, as indicated by the smaller standard deviations. It was also observed that the mean value of the MTR asymmetry was closer to that of the GT, indicating improved accuracy of the results.

### 3.4. Results of Iopamidol Phantom Case

**Figure 10:**
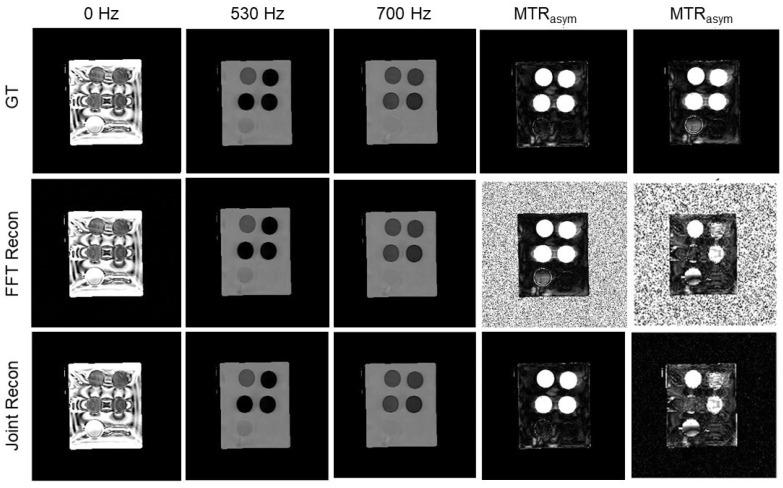
GT MR images, MR images reconstructed by the FFT-based algorithm and our algorithm, and MTR asymmetry map at $\omega_{2}$ = 530 Hz and $\omega_{3}$ = 700 Hz of the iopamidol phantom with noise level 15% at offset frequencies 0, 530, and 700 Hz. Image windows at 0 Hz are [0 0.35] and [0.5 1.5] at 530∼700 Hz. MTR asymmetry map window is [0 0.5].

Moving on to a more challenging case of the multi-compartment iopamidol phantom, which was reconstructed with three components in the Z-spectrum, the results for the 15% noise level are shown in Figure 10. For different noise levels of 0.5∼40%, the averaged SNRs for our algorithm were 11.5 to 71.6 dB, in contrast to those of the conventional algorithm (9.2∼46.2 dB).

**Figure 11:**
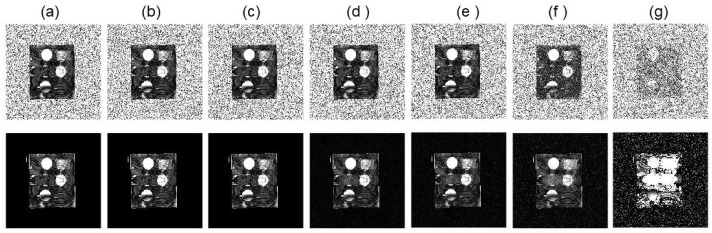
MTR asymmetry maps at $\omega_{3}$ = 700 Hz of the conventional algorithm (upper row) and our algorithm (lower row) of the experimental iopamidol phantom with various noise levels (**a**) 0.5%, (**b**) 2.5%, (**c**) 4.5%, (**d**) 10.5%, (**e**) 15%, (**f**) 25%, and (**g**) 40%, respectively. MTR asymmetry map window is [0 0.5].

**Figure 12:**
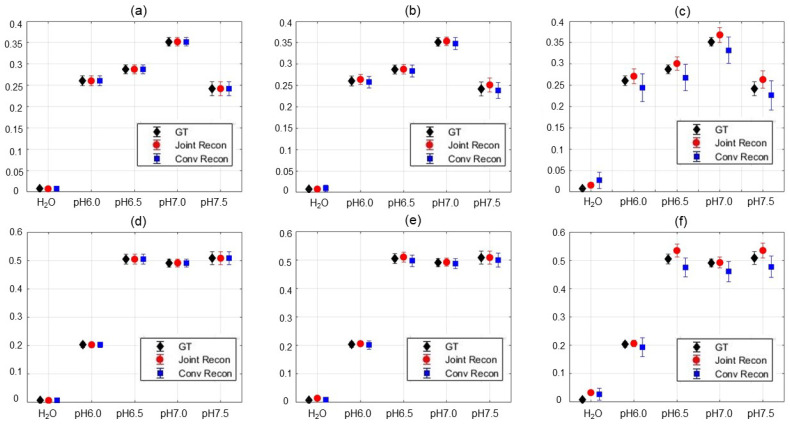
Mean and standard deviation of MTR asymmetry in each insert at (**a**–**c**) $\omega_{2}$ = 530 Hz and (**d**–**f**) $\omega_{3}$ = 700 Hz for water and iopamidol solutions of various pH values 6.0, 6.5, 7.0, and 7.5 of the GT; results reconstructed by the conventional method and our method with noise levels of 0.5%, 4.5%, and 10.5%, respectively.

The MTR asymmetry maps shown in Figure 11a–h demonstrated again the improvement in the prediction of the Z-spectrum MTR asymmetry analysis for each pixel in the iopamidol phantom using the joint reconstruction approach. Similar to the previous case, we present in Figure 12 the mean and standard deviation of MTR asymmetry at 530 Hz and 700 Hz for different ROIs of various pH values 6.0, 6.5, 7.0, and 7.5 at noise levels of 0.5∼10.5%. A similar behavior was observed as in the previous choline case. The advantages of our algorithm appeared to be more obvious in the high noise level situation, as indicated by the reduction of standard deviation and slightly increased accuracy.

### 3.5. Reduction in Number of Offset Frequencies

**Figure 13:**
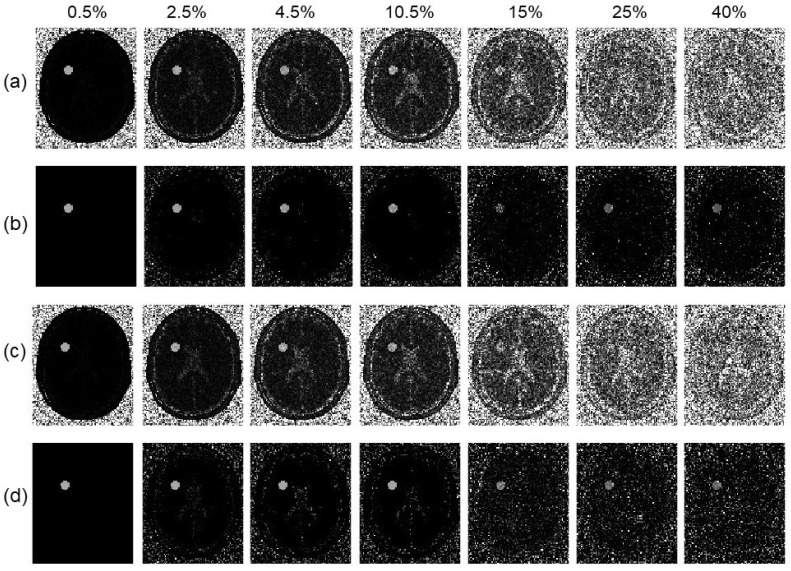
MTR asymmetry maps at $\omega_{2}$ = 450 Hz from the conventional algorithm (**a**,**c**) and our algorithm (**b**,**d**) for the simulated phantom with (**a**,**b**) 15.6% and (**c**,**d**) 27.5% reduction in offset frequencies. MTR asymmetry map window is [0 0.2].

As for the scenarios with reduced numbers of frequencies, the MTR asymmetry map results for the simulated phantom with 15.6% and 27.5% reductions with respect to the original 51% offset frequencies are shown in Figure 13. As expected, the MTR asymmetry map became noisier, as noise amplitude in the k-space data was increased. In both scenarios with different reductions in the number of offset frequencies, our method achieved better performance than that of the conventional method. In particular, in the case with a 27.5% reduction in offset frequencies at the 40% noise level, the MTR asymmetry at ROI can still be visually identified, whereas this was not possible using the conventional algorithm due to substantially increased background noise.

## 4. Discussion

A key component in our algorithm is the incorporation of the correlation of solutions along the spatial dimension via the BM3D approach and that along the offset frequency dimension via the curve fitting approach. Yet the reconstruction algorithm presented in Equations (8)∼(15) can be viewed as a general form, rather than specifically relying on these two regularization approaches. In fact, other image domain or frequency domain regularization terms can be naturally incorporated in this algorithm to replace the ones chosen here. In this paper, we chose those relatively simple classical regularization approaches to illustrate our idea and demonstrate the validity of our method. With more advanced regularization approaches, we expect the performance of the algorithm to be further enhanced. In particular, in recent years, deep learning (DL)-based regularization methods have been extensively studied and neural networks can be trained to understand the desired image properties and ensure them in the solutions [27,38]. These regularization methods can certainly be incorporated in our algorithm. There are also end-to-end DL-based reconstruction approaches that enable direct mapping from the measurement space to the solution space. Compared to these approaches, the structure of our algorithm holds the advantage of model interpretability, a key feature desired for clinical applications of deep learning algorithms [39]. Additionally, in the current study with the classical regularization terms incorporated, we avoid the limitation of data-driven DL approaches, such as dependence on data quality for training and model interference, robustness, etc. [27].

One particular reason for the enhanced performance of our algorithm was the utilization of images at all offset frequencies to help enforce solution quality. The detailed updating formula through each iteration used to reconstruct CEST MR images at various offset frequencies is shown in Equations (8) and (9). In these formulas, when generating a new solution for a frequency ω in the iterative process, the solution was produced not only by incorporating the condition specified by the data fidelity term of the frequency, but also the information from images at all other frequencies from the previous iteration step. In this way, the noise in an image at one frequency was effectively averaged out by taking advantage of images at other frequencies, yielding enhanced quality of the reconstructed images, as compared to the conventional reconstruction method that approaches MR images at each frequency individually.

Parameter selection is always an important topic for image reconstruction algorithms, as it critically affects practicality of the algorithms. In the model Equation (7), there is only one model parameter, namely the regularization term strength λ. Because of the interpretable nature of our algorithm that viewed the relevant step in Equation (11) as a denoising step, this parameter was adaptively estimated during the iterative process related to the noise level of the intermediate solution. We purposely reduced the estimated parameter value in this study to avoid over-smoothing the solution. On the reconstruction algorithm side, the ADMM algorithm further introduced a parameter ρ. This parameter is not expected to impact the reconstruction results at convergence, but only the rate and stability of convergence. We empirically found a suitable parameter value ρ=0.5 to achieve a reasonable convergence rate.

There are several limitations in our work. First, the theoretical convergence of the reconstruction approach is not guaranteed. Although we employed the ADMM approach to allow us to split the reconstruction approach into a set of problems, each focusing on one aspect of the solution, we heuristically introduced an image denoising step in the spatial domain and data fitting step along the frequency domain, making it hard to justify convergence. Yet, based on our numerical experiments, we observed the convergence behavior of the algorithm. Second, despite the three cases presented in the paper, further validation studies are needed to comprehensively assess the advantages and limitations of the algorithm. In particular, the simulation and semi-experimental studies presented in this paper may not be able to fully capture the challenges in real CEST MRI studies. The algorithms may not adequately consider the physics in CEST MRI data acquisition. Hence, the current study only serves as a proof-of-principle investigation of our idea, and future studies are being designed. Along the same vein, it is necessary to further perform a validation study with in vivo data. At present, we do not have access to in vivo data, and we are actively pursuing this direction. We believe that the results obtained with the current study set a strong foundation for the next steps. Results of in vivo studies will be presented in future publications. Third, as mentioned previously, the proposed algorithm is a general framework that can incorporate other types of regularization terms in spatial and frequency domain. Hence, one limitation of the present work is that we only compared it with the conventional FFT-based reconstruction, whereas more advanced DL-based reconstruction was not studied. Future work will include comparison studies with these algorithms.

## 5. Conclusions

Aiming at reconstructing CEST MRI images with noisy or sparse measurements along the frequency dimension, hence reducing acquisition time, in this study we explored image correlations along the spatial and Z-spectral frequency dimension via a joint *k*-ω reconstruction model. We formulate the reconstruction model as an optimization problem with respect to MR images at all frequencies ω and incorporating regularizations along the spatial and frequency dimensions. The optimization problem was solved using the ADMM. Evaluated on a simulated CEST MRI phantom and choline and iopamidol phantoms semi-experimentally with added Gaussian noise demonstrated potential advantages of our algorithm compared to the conventional approach in terms of improving SNR of the reconstructed images and reducing variations in MTR asymmetry, as well as allowing the reduction of number of frequencies. In the choline and iopamidol phantom cases with 10.5% noise in the measurement data, our method achieved an averaged SNR of 31.0 dB and 32.2 dB, compared to a SNR of 24.7 dB and 24.4 dB in the conventional reconstruction approach. It reduced uncertainty of the estimation of MTR asymmetry over all regions of interest by 54.4% and 43.7% in the two cases, from 1.71 and 2.38 to 0.78 and 1.71, respectively.

## Data Availability

The raw data supporting the conclusions of this article will be made available by the authors on request.
